# Tack-only fixation of lumen-apposing metal stents: leave the suture at home

**DOI:** 10.1016/j.vgie.2025.05.007

**Published:** 2025-06-02

**Authors:** Yara Salameh, Hadi K. Abou Zeid, Kamal Abi Mosleh, Andrew C. Storm

**Affiliations:** 1 Division of Gastroenterology and Hepatology, Mayo Clinic, Rochester, Minnesota, USA; 2 Department of Surgery, Division of Metabolic and Abdominal Wall Reconstructive Surgery, Mayo Clinic, Rochester, Minnesota, USA

## Abstract

**Background and Aims:**

Roux-en-Y gastric bypass (RYGB) may result in refractory gastrojejunal anastomosis (GJA) strictures, which are sometimes treated with lumen-apposing metal stents (LAMSs). To prevent premature stent migration, its fixation may be considered. A tack-and-suture device, designed for through-the-scope mucosal closure, deploys a suture with 4 helical tacks that can also serve in stent fixation. We present a novel tack-only technique for LAMS fixation at the GJA for post-RYGB cases of GJA stenosis.

**Methods:**

Four patients who underwent RYGB with GJA stenosis underwent LAMS placement fixated using a tack-only approach. The suture was removed ex vivo, and individual tacks were advanced and drilled through the mesh of the LAMSs’ proximal flange at multiple sites to anchor the stent.

**Results:**

All 4 LAMSs were successfully fixated without adverse events. The stents remained in place beyond 8.6 weeks. One was removed endoscopically, and 2 were passed spontaneously, with all 3 patients showing resolution of stenosis and symptoms. The fourth patient has been asymptomatic with the stent still in place at 20-week follow-up, with removal planned at 24 weeks.

**Conclusions:**

This pilot study suggests the potential feasibility and safety of a tack-only LAMS fixation technique at the GJA. Larger studies are needed to validate this approach.

## Introduction

Refractory gastrojejunal anastomosis (GJA) strictures after Roux-en-Y gastric bypass often are treated with endoscopic stenting. Lumen-apposing metal stents (LAMSs) are suitable for this site because of their dumbbell-shaped design.^1^ Although manufacturers advertise a dwell time of 8.6 weeks^2^ for LAMSs, real-world cohort studies have reported migration rates ranging from 14%^3^ to 27.3%.^4^ Factors predisposing to LAMS displacement include narrow or angulated anatomy and stricture dilation and remodeling over time.^5^ Thus, clinicians may perform stent fixation to prevent migration, aiming for longer dwell times.^3^

**Figure 1:**
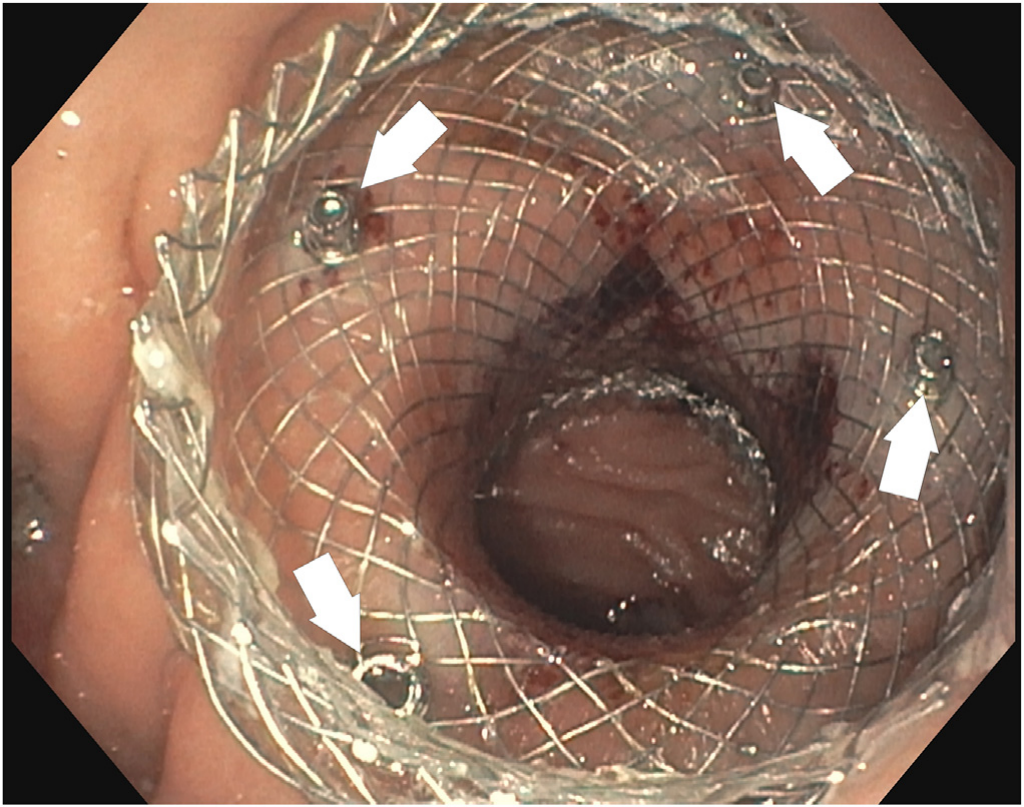
A total of 4 tacks (*white arrows*) are placed to anchor the lumen-apposing metal stent at a gastrojejunal anastomosis.

The use of a tack-and-suture device is documented for anchoring stents, including LAMSs.^6^^,^^7^ This technique involves 4 surgical-steel helical tacks, a running suture, and a cinch. These tacks resemble screw-type fixation anchors for mesh fixation in hernia repair surgeries. LAMSs, having mesh-like walls in a tighter weave pattern, are particularly suitable for tack fixation, and may not require a suture or cinch. In this video case report (Video 1, available online at www.videogie.org), we present an unexplored use of X-Tack (Boston Scientific, Marlborough, Mass, USA) involving tack-only fixation of LAMSs for GJA strictures (Fig. 1).

## Cases

Our case series included 4 patients who underwent RYGB. Institutional review board approval was obtained from Mayo Clinic (Rochester, Minn, USA) on January 23, 2023 (protocol number 22-011344). Informed consent was obtained at the time of procedure for use of materials for academic purposes including the publication of case details. All patients presented with obstructive symptoms. On endoscopy, all were also found to have a severe GJA stenosis that could not be traversed. We opted to stent the area because of previously failed dilations in 1 case, the severity of the stenosis and the patient's long distance from home in 2 cases, and because of the concomitant presence of a marginal ulcer causing a leak in the final case (Table 1).

**Table 1:** Summary of LAMS fixation cases across a stenosed gastrojejunal anastomosis using a tack-only technique

Case number	Age, y	Presenting symptoms	Stenosis size, mm	Sex	LAMS size, mm	Dilation of stenosis, final diameter, mm	Number of tacks placed	AE	Total dwell time, wk
1	31	Dysphagia, PO intolerance	“Severe”	F	15 × 10	No	3	No	13.6
2	68	Vomiting	“Severe”	M	20 × 15	18	4	No	22.1
3	45	Vomiting, chest and abdominal pain	4	F	15 × 10	12	3	No	10.1
4	46	Weakness, abdominal pain	4	M	15 × 10	No	4	No	10.6

*AE*, Adverse event; *F*, female; *LAMS*, lumen-apposing metal stent, *M*, male; *PO*, peroral.

## Procedure

The LAMS was deployed across the anastomosis with its proximal flange within the gastric pouch and the distal flange within the proximal jejunum. Its ideal position was confirmed by contrast flowing easily through the anastomosis, allowing for fixation maneuvers to proceed.

**Figure 2:**
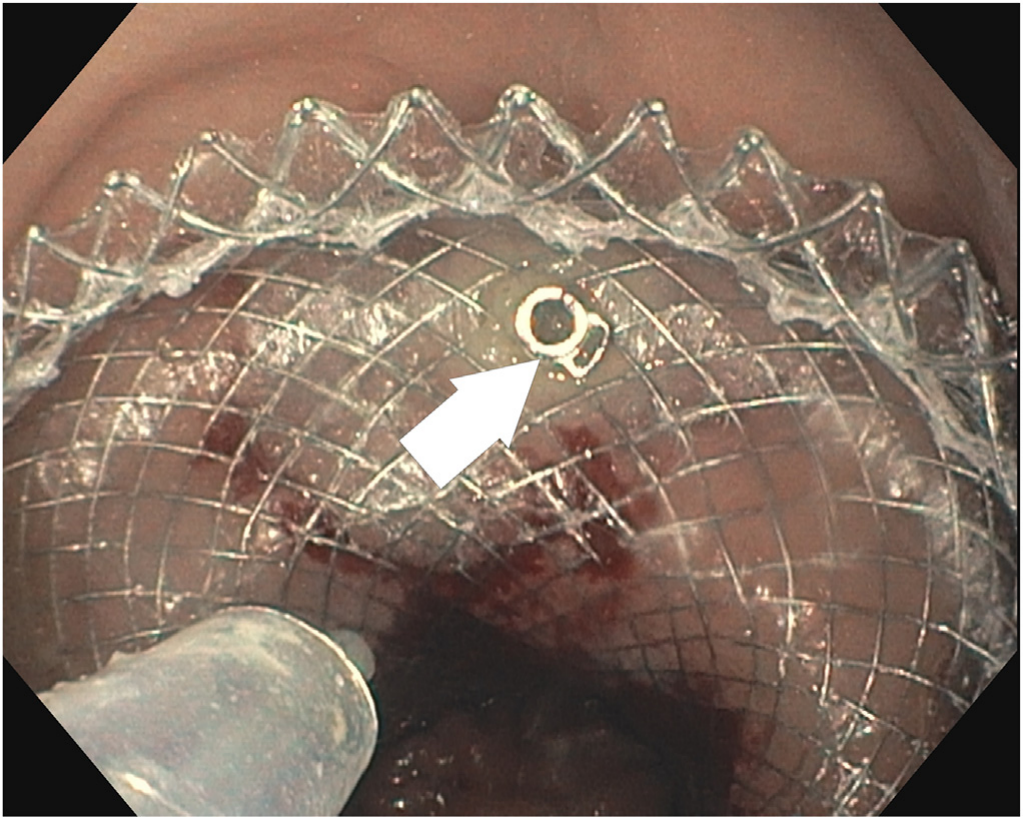
First tack (*white arrow*) is drilled and deployed through the lumen-apposing metal stent into adjacent mucosa.

**Figure 3:**
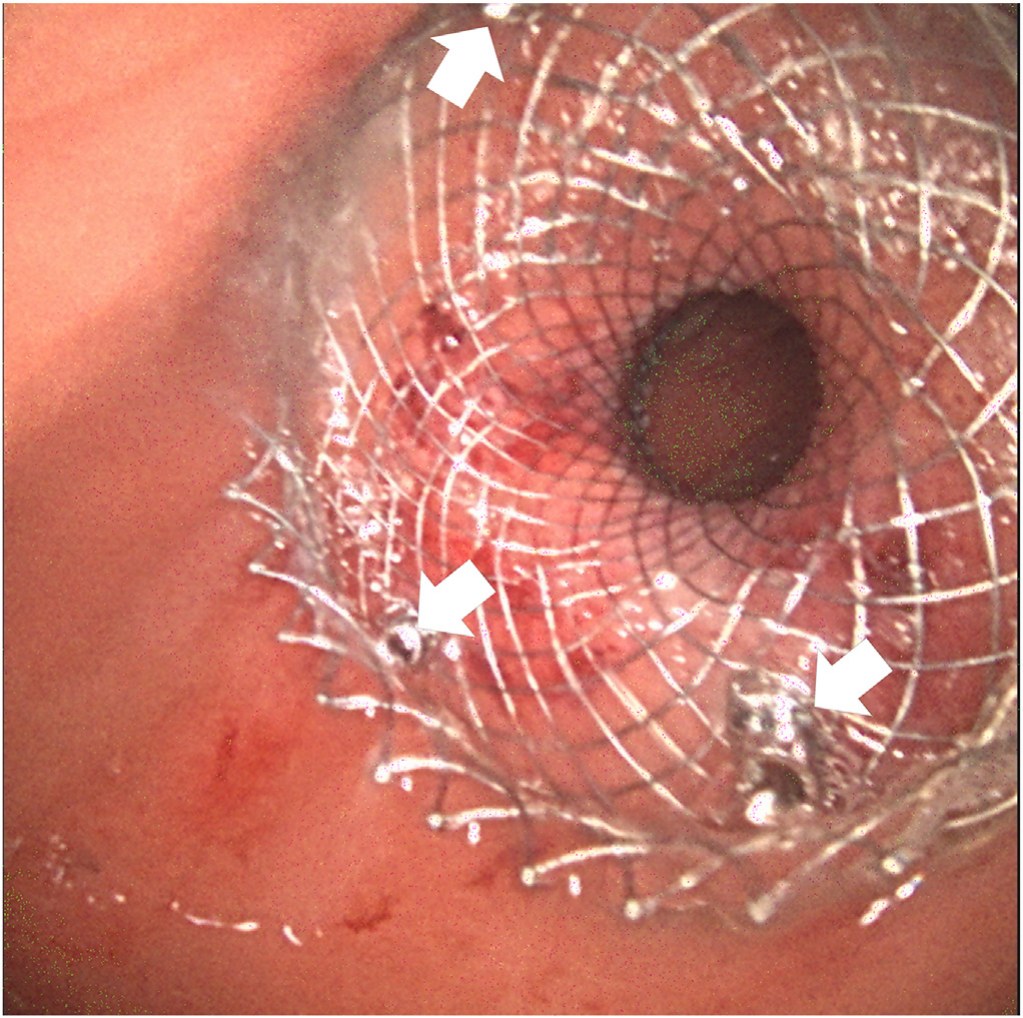
A total of 3 tacks (*white arrows*) are placed to anchor the lumen-apposing metal stent at a gastrojejunal anastomosis.

Before introducing the tack-and-suture device into the scope's working channel, the suture tethered to the 4 tacks is removed by cutting the 3-0 polypropylene suture. Each tack is loaded 1 by 1 onto the tack-driver catheter as per usual recommended use. After introducing the device, the first tack is advanced through the stent's weave structure, drilled into the healthy apposing target tissue, and expelled from the tack-driver catheter (Fig. 2). The tack eyelet, which previously held the suture, serves as a stop to prevent overadvancement of the tack through the stent weave. Subsequent tacks are deployed in the same fashion, aiming for unique areas of the proximal flange. Two patients had 4 tacks placed, with 1 in each quadrant (Fig. 1). The remaining 2 patients had 3 tacks placed (Fig. 3) as determined by the endoscopist's preference.

## Outcome

All stents were successfully and safely fixated with no immediate adverse events. Three patients were discharged on the day of procedure, whereas the patient who had a concomitant leak was admitted for continuation of care. All the LAMSs remained in place beyond 8.6 weeks as recommended by the manufacturers' indication for use without premature migration. Dwell time was defined as duration of stent therapy from placement until endoscopic removal, or evidence of migration on radiography or endoscopic imaging.

**Figure 4:**
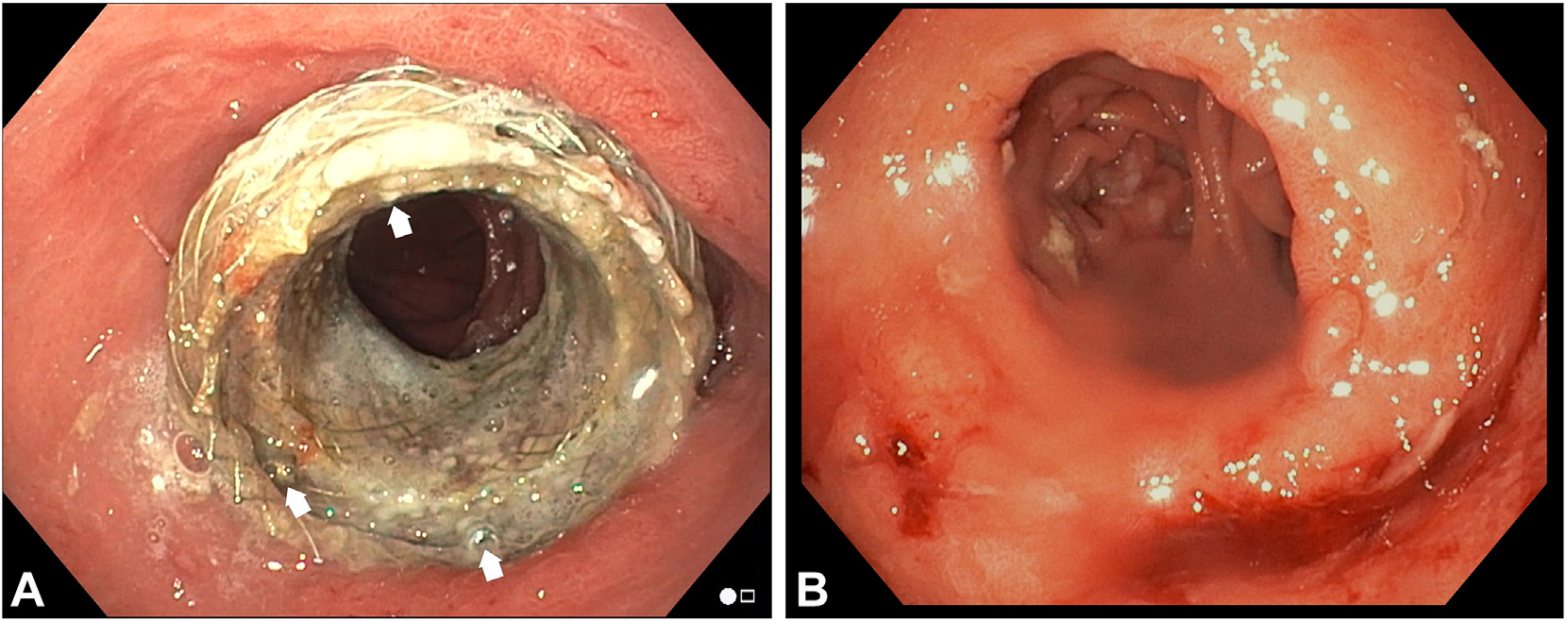
**A,** Follow-up endoscopy 3 months after lumen-apposing metal stent placement and fixation using 3 tacks showing all tacks remaining intact (*white arrows*) and the stent in stable position. **B,** Gastrojejunal anastomosis after lumen-apposing metal stent and removal of helical tacks.

In 1 case, the intact stent with tacks was endoscopically removed at 13.6 weeks as the stenosis resolved (Fig. 4A and B). Stent removal is achieved by applying a reverse traction with therapeutic grasping forceps. This force is transferred to the helical steel tacks, causing them to elongate and detach from the tissue. Two stents were eventually excreted by the patients. In those cases, there was resolution of the stenosis and its associated symptoms, as confirmed by endoscopy and imaging. The final patient had follow-up imaging 20 weeks after fixation, confirming fixed placement of the LAMS with tacks. This patient did not report any symptoms and has not yet undergone stent removal, planned for 24 weeks. In all cases with confirmed stent removal or migration, no retained tacks were visualized radiographically or endoscopically.

## Discussion

In a retrospective study, Mahmoud et al^4^ evaluated 128 LAMS placements for benign strictures and reported an overall migration rate of 27.3%. Among the 107 stents placed at anastomotic strictures, including 60 at GJAs, the migration rate was 27.1%. Although tight strictures may initially reduce migration, this real-world cohort shows that stricture remodeling over time still poses a significant risk, highlighting the potential need for adjunctive LAMS fixation.

Both over-the-scope clips and endoscopic suturing are effective for stent fixation, with a meta-analysis by Gangwani et al^8^ showing no significant difference in migration rates. However, both require advanced expertise and longer procedural times.^8^ The efficacy of over-the-scope clips is contingent on adequate tissue pliability, often compromised in fibrotic or stenotic regions.^9^ Likewise, shallow or overtightened sutures may compromise fixation.^10^ These limitations have driven interest in novel fixation techniques.

This case series is the first to describe a tack-only approach using a through-the-scope tack-and-suture device for LAMS fixation. Law et al^10^ highlight that the device's helical grasper enables consistent submucosal engagement. The tack-only method avoids risks associated with cinching and suture overtightening and preliminarily demonstrates no tack-related adverse events during follow-up. Its favorable safety and efficacy may stem from both the submucosal anchoring mechanism and the controlled, stepwise deployment, which allows precise placement while avoiding fragile tissue and major vessels.

## Conclusions

Our pilot experience with a novel tack-only technique for LAMS fixation at the GJA suggests its potential safety and effectiveness, permitting extended stent dwell time. Although promising, this technique remains preliminary; further studies are needed to validate this technique and explore applicability in LAMS fixation settings.

## Patient consent

Written informed consent was obtained from the patients for publication of this report and any accompanying video or images.

## Disclosure

The following authors disclosed financial relationships: A. Storm: recipient of research grants from Apollo Endosurgery, 10.13039/100008497Boston Scientific, Endogenex, Endo-TAGSS, Envision Endoscopy, Enterasense, MGI Medical, OnePass, and SofTac and consultant for Ambu, 10.13039/100008497Boston Scientific, 10.13039/100010479Cook Medical, 10.13039/501100021563Intuitive, 10.13039/100004374Medtronic, and Olympus. All other authors disclosed no financial relationships.

## Supplementary data

Video 1 Technique for a novel use of a tack-only approach for lumen-apposing metal stent fixation at a gastrojejunal anastomosis.
